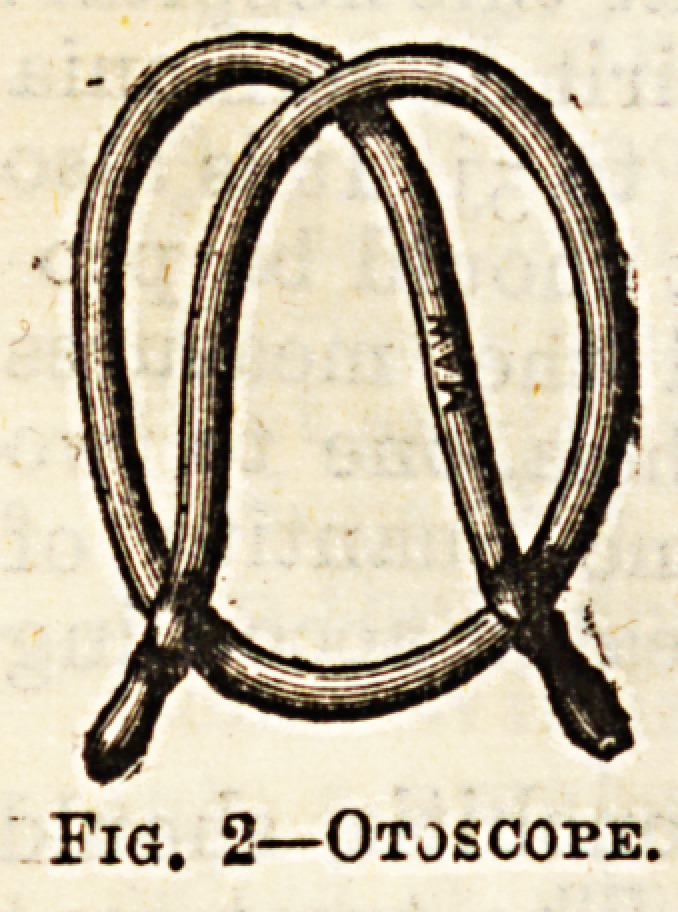# The Treatment of Some of the Complications and Sequelæ of Middle Ear Suppuration

**Published:** 1892-12-17

**Authors:** 


### ROYAL INFIRMARY, EDINBURGH.

### Treatment of Some of the Complications and Sequelæ of Middle Ear Suppuration.

In a recent article we considered at some length the treatment of chronic suppuration of the middle ear as practised in this school. Accompanying and following this common ailment there are various complications and sequelæ which now claim our attention.

1. *Deafness.*—In all cases in which there has been long continued suppuration from the tympanum the membrane is of necessity perforated, and more frequently than not the aperture shows no tendency to close even after the discharge has ceased. The result is considerable impairment of hearing. Fortunately much benefit is to be derived from the use of an artificial membrane, or to speak more accurately, of some appliance which will take the place of the membrane in so far as it supports the auditory ossicles and transmits vibrations to them. The simplest, cheapest, and probably the best of these is a thin plug of absorbent wool, about one inch long, moistened in glycerine or water; or should there still be discharge from the ear, in a solution of boracic acid or sulphate of zinc. This plug is placed against the perforation in the membrane, with the aid of a pair of forceps. The plug is changed daily, is not worn at night, and at first should only be used for an hour, or even less, at a time, till the patient gets accustomed to its presence. It is needless to add that the ear must be kept scrupulously clean, the syringe being regularly employed.

In some cases the indrawing of the membrane which results on the cessation of the suppuration requires to be counteracted by inflating the tympanum through he Eustachian tube. For this purpose the Eustachian

Fig. 1.—Eustachian Catheter.

catheter (Fig. 1) is employed. This is made of gum elastic, is slightly curved at the point, and has a wide mouth, into which the nozzle of Politzer's bag fits. It is passed along the floor of the nostril, the point being directed downwards, till it strikes the posterior wall of the pharynx. The point is then turned towards the middle line, and the instrument withdrawn till it hitches on the nasal septum. If the point be now rotated through half a circle it will come to lie directly opposite the opening of the Eustachian tube. The passage of the catheter is facilitated if the point of the patient's nose be slightly raised with the thumb of the surgeon's left hand. When the catheter is in position, the nozzle of the Politzer's air bag is fitted into it, and a current of air thrown in. It is found most convenient to hold the air bag in the left armpit while the catheter is being passed. The patient is usually able to tell if the air has inflated the tympanum satisfactorily or not by his sensations, but should the surgeon wish to make certain he can do so by using the otoscope (Fig. 2), a simple india-rubber tube fitted with an ear-piece at each end. One end is placed in the affected ear of the patient, and the other in the surgeon's ear, and in this way the free entrance of air, or the reverse, can be ascertained.

Fig. 2—Otoscope.

2. *Mastoid Disease.*—The air cells of the mastoid process being in direct continuity with the middle ear, it is not unnatural that when pus is pent up in the latter it should find its way back into the former and there set up mischief. As a matter of fact, in a considerable number of cases, middle ear suppuration is followed by more grave symptoms, indicative of inflammation and the formation of pus in the mastoid cells. These are general feverishness with high temperature, possibly preceded by rigors, rapid pulse and associated gastric disturbance. The patient complains of deep-seated throbbing pain behind the ear, and tenderness on percussion in this region. If the inflammation be confined to the internal lining of the cells no other important symptoms are manifest, but if it has spread, as it very frequently does, to the periosteum outside, there are in addition redness and swelling over the mastoid process, the lobe of the ear being carried out from the head.

The first point to attend to in treatment is to make certain that no obstruction to the escape of pus exists in the meatus, and whether or not frequent douching with boracic lotion must be employed. To relieve tension a free incision down to the bone is made over the mastoid process. To avoid damaging the posterior auricular artery this incision is made half an inch behind the lobe of the ear, and parallel with its attachment to the skull; and it should be made from below upwards lest the knife slip off the bone in among the soft structures of the neck.

Not unfrequently the bone is found bare, and a probe will often detect a small opening passing into the air cells. With the aid of a small gouge and mallet this opening is enlarged, and the contents cleared out with a sharp spoon and a current of lotion. Should no fistula be found, an opening is made with the gouge slightly above and behind the external auditory meatus, in a direction forwards and inwards to avoid the lateral sinus. This opening has to be kept patent for some time by means of a lead drainage tube or plug, so that the ear and mastoid cells may be frequently cleared out by a stream of lotion flowing in through one and out through the other. When all evidences of inflammation and suppuration have subsided, the plug may be removed and the wound allowed to close up.

3. *Cerebral and Cerebellar Abscess.*—These conditions frequently follow on middle ear suppuration which has spread to the mastoid cells. The symptoms manifested by the patient, in addition to those of the antecedent condition, are rigors, followed by subnormal temperature—97° F., with a downward tendency; slow pulse, 65-55, or even slower, constipation, and occasional vomiting. At the same time the patient becomes dull and apathetic, answering questions intelligently, although very unwillingly. More grave symptoms may supervene and terminate fatally.

The only rational treatment is to open the cranium and evacuate the pus. When in the brain the abscess is usually in the tempero-spheroidal lobe, and the seat of opening, made with a gouge, is about one inch above and behind the middle of the external auditory meatus.

If the cerebellum be the seat of the pus, then the trephine is applied just below the inferior curved line on the occipital bone. On exposing the brain, should pus not flow, an exploring needle, most carefully purified, is passed in various directions into the brain substance till the abscess is struck. The opening is enlarged by a director and dressing forceps, and a drainage tube inserted. An antiseptic dressing completes the procedure.

4. *Septic Meningitis*, indicated by intense general headache, short, sharp cries, and high temperature, can only be treated palliatively by leeches, the ice bag, and opium to relieve the agony of the patient.

5. *Phlebitis* of the lateral sinus or jugular vein is usually the precursor of geneal pyæmia and death, to prevent which every effort must be made to clean out the ears and mastoid cells—the source of the mischief; to stimulate the patient with brandy, ammonia, strophanthus, &c.; and to counteract the septic element by the administration of internal antiseptics, such as quinine, sulpho-carbonate of soda, iron, &c.

6. *Caries and Necrosis* of bone is treated by the removal, when possible, of the affected bone, in addition to general treatment.

## Figures and Tables

**Fig. 1. f1:**
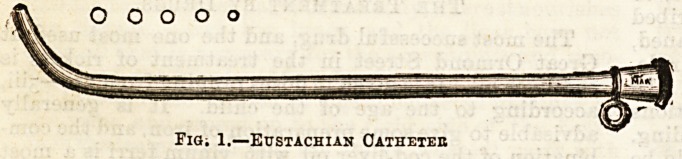


**Fig. 2 f2:**